# Double-Network Polymer Electrolytes with Ionic Liquids for Lithium Metal Batteries

**DOI:** 10.3390/polym14173435

**Published:** 2022-08-23

**Authors:** Chenjing Zhu, Yi Ning, Yizhi Jiang, Guangji Li, Qiwei Pan

**Affiliations:** 1 School of Materials Science and Engineering, South China University of Technology, Guangzhou 510640, China; 2 Key Laboratory of Polymer Processing Engineering, South China University of Technology, Ministry of Education, Guangzhou 510640, China; 3 Guangdong Provincial Key Enterprise Laboratory of Novel Polyamide 6 Functional Fiber Materials Research and Application, Jiangmen 529100, China

**Keywords:** double-network polymer, ionic liquid, composite polymer electrolyte, lithium metal batteries, lithium dendrites

## Abstract

Solid-state polymer electrolytes have become promising candidates for high-energy-density lithium metal batteries (LMBs). However, they suffer from low ionic conductivities at room temperature. In this work, two types of composite polymer electrolytes based on a double-network polymer, an ionic liquid (IL) of 1-butyl-1-methylpyrrolidinium bis(trifluoromethanesulfonyl) imide (Pyr_14_TFSI) or 1-ethyl-3-methylimidazolium bis((trifluoromethyl)sulfonyl) imide (EmimTFSI), and bis(trifluoromethane)sulfonamide lithium salt (LiTFSI) were prepared by a facile one-pot method. The two types of CPEs possess good mechanical properties, excellent thermal stability, and high ionic conductivities greater than 10^−4^ S cm^−1^ at 20 °C with 26 wt% IL. The performance diversity of the CPEs was also carefully investigated through a series of electrochemical measurements. Although the CPEs containing EmimTFSI show higher ionic conductivities than those of CPEs with Pyr_14_TFSI, the latter ones have wider electrochemical stability windows and better resistance to the growth of lithium dendrites. Moreover, CPE with 34 wt% Pyr_14_TFSI leads to Li/LiFePO_4_ batteries with favorable rate capability and cycling stability and a columbic efficiency of 98.8% at 20 °C, which suggests that CPEs are promising for practical application in solid-state LMBs.

## 1. Introduction

Lithium metal shows an ultralow reduction potential (−3.4 V) and high specific capacity (3860 mAh g^−1^), which can match lithiated or unlithiated cathodes, such as LiFePO_4_, sulfur, oxygen, etc., making it a hotspot in superpower and energy storage devices [1,2]. However, complex and continuous side reactions between lithium metal and traditional liquid electrolytes cause serious interface problems due to the high activity of lithium metal [3,4]. The intrinsic safety hazards and the internal short circuit caused by the uncontrollable growth of lithium dendrites have become bottlenecks restricting the application of lithium anodes [5,6,7,8].

Solid polymer electrolytes (SPEs) are promising candidates for lithium anodes because of their excellent comprehensive properties, such as high mechanical strength, excellent thermal stability, and favorable electrochemical properties, which allow them to effectively inhibit the growth of lithium dendrites [9,10,11,12]. An obstacle to its application is the relatively low ionic conductivities at room temperature related to the thermodynamic characteristics of the polymer segment [13]. To date, poly (ethylene oxide) (PEO)-based hybrid electrolytes and cross-linked PEO have been extensively reported to overcome this drawback of SPEs [14,15,16,17]. Inorganic nanoparticles or network structures can significantly reduce the crystallinity of PEO, thus improving the ionic conductivities without damaging the mechanical properties. However, few SPEs meet the practical application requirements of lithium metal batteries (LMBs) at room temperature.

Ionic liquids (ILs) represent an interesting class of room-temperature fluids, whose organic cations are mainly imidazolium, pyrrolidinium, piperidinium, etc., and the anions (TFSI^−^, BF_4_^−^, FSI^−^, etc.) can be obtained through ion-exchange reactions with different electrolytes [18,19]. Based on their unique structures, ILs have high ionic conductivities (up to 10^−3^ S cm^−1^) at room temperature, excellent thermal stability, low vapor pressure, and a wide electrochemical stability window. They have been widely studied and applied since the 20th century [20,21]. When used as additives or solvents, ILs integrated with polymers form composite or gel polymer electrolytes (CPEs or GPEs) with excellent performance [20,22,23]. Li et al. reported a new type of gel polymer electrolyte (GPE) where the ionic liquid is introduced into adjustable POSS-PEO networks [24]. GPEs show an excellent ability to inhibit the growth of lithium dendrites, and the corresponding Li/LiFePO_4_ batteries exhibit outstanding cycling stability and coulombic efficiency in a wide temperature range of 0–90 °C, which has great potential for the application of LMBs. In addition, some studies have introduced inorganic fillers to IL-based polymer electrolytes to compensate for the loss of mechanical strength due to the addition of ILs. Reported fillers include inert ones, such as sepiolite [25] and nanocellulose [26], and the lithium-conducting particles LLZTO [27]. Zhang et al. reported a thin and flexible membrane of a composite polymer electrolyte (CPE) that contained a lithium-conducting Li_1.3_Al_0.3_Ti_1.7_(PO_4_)_3_ filler, PEO matrix, and IL [28]. The synergistic effect of the organic–inorganic hybrid system endows CPE films with a stable interface with lithium metal and leads to the superior rate capability of the corresponding batteries.

Currently, the high cost of GPEs with a large amount of ionic liquid limits their practical application. It is also challenging for researchers to determine the appropriate ratio of polymer/ILs/fillers in composite electrolytes and explain the corresponding mechanism [29]. Therefore, realizing the practical application of ionic GPEs with a facile synthetic method, low cost, and excellent comprehensive performance in LMBs at room or lower temperature is still a significant issue.

Single-network polymer electrolytes, semi-interpenetrating network polymer electrolytes, or CPEs based on these networks and ILs have been demonstrated to be superior in LMBs [5,30,31,32]. A double-network (DN) polymer is a cross-linked polymer with two interpenetrated but individual polymer networks and is first designed for hydrogels with super-high toughness [33,34]. Compared to single-network hydrogels, DN hydrogels show improved mechanical properties and more flexibility in molecular design since networks with different functions can be introduced into the DN polymer. However, DN polymers are seldom used for polymer electrolytes. In this work, PEO-based CPEs with a DN structure and different ionic liquids were synthesized by a facile one-pot method to achieve room-temperature solid-state LMBs. CPEs with different amounts of IL, 1-ethyl-3-methylimidazolium bis(trifluoromethylsulfonyl) imide (EmimTFSI), or 1-butyl-1-methylpyrrolidinium bis(trifluoromethylsulfonyl) imide (Pyr_14_TFSI) were thoroughly characterized and compared. The ionic conductivity of the DN polymer electrolyte was greatly improved when incorporated with the IL, and it reached 10^−3^ S/cm with high IL content at 20 °C. The effects of the amount and type of IL on the lithium transference number, electrochemical stability window, resistance to the growth of lithium dendrites, mechanical properties, and Li/LiFePO_4_ battery performance were also studied.

## 2. Experimental Section

### 2.1. Materials

Poly(ethylene glycol) bis(3-aminopropyl) terminated (*M*_n_ = 2257 g mol^−1^, confirmed by ^1^HNMR, denoted as PEG2K), 1-ethyl-3-methylimidazolium bis((trifluoromethyl)sulfonyl)imide (EmimTFSI, 99%), and 1-butyl-1-methylpyrrolidinium bis(trifluoromethanesulfonyl)imide (Pyr_14_TFSI, 99%) were all purchased from Acros. Bis(trifluoromethane)sulfonamide lithium salt (LiTFSI, 99.95%);was from Aldrich. Trimethylolpropane triglycidyl ether (99%) was obtained from Damsas-beta. Poly(ethylene glycol) methyl ether acrylate (*M*_n_ = 480 g·mol^−1^, PEGMEA) and poly(ethylene glycol) diacrylate (*M*_n_ ≈ 600 g·mol^−1^, PEGDA) were obtained from Aladdin, where the inhibitor was removed before use. The initiator dicumyl peroxide (DCP, Aladdin) was recrystallized before use. LiFePO_4_ and carbon black were supplied by Ruiheda Co., Ltd., ShenZhen (ShenZhen, China). Lithium foil (700 μm) was from China Energy Lithium Co., Ltd. (Tianjin, China).

### 2.2. Preparation of Composite Polymer Electrolytes

**Scheme 1:**
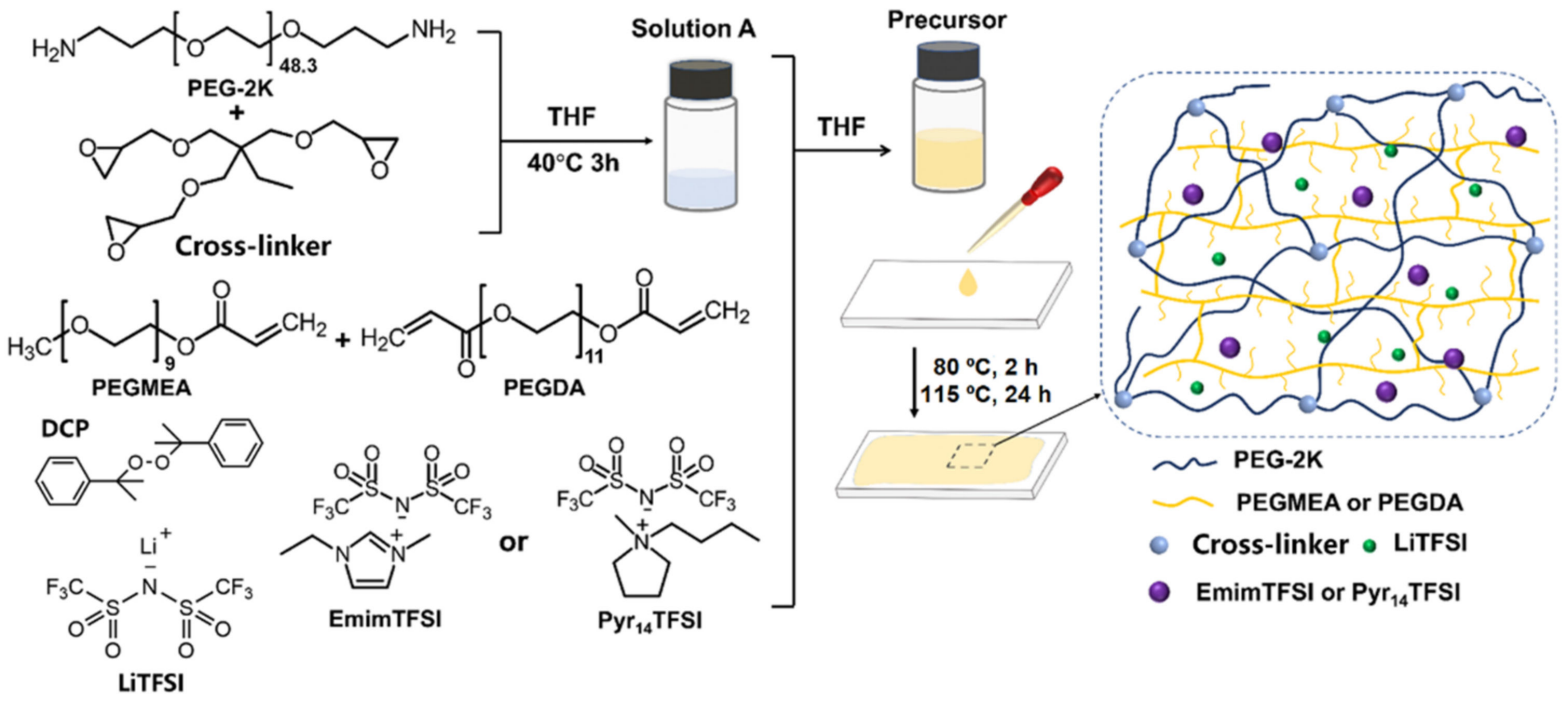
The synthetic route and ideal double-network CPEs.

The synthetic route of CPEs is shown in Scheme 1. PEG2K (76.4 mg) and trimethylolpropane triglycidyl ether (13.6 mg, molar ratio = 3:4) were dissolved in 0.5 mL of THF and reacted at 40 °C for 3 h to obtain precursor A. PEGMEA (89.45 mg) and PEGDA (0.55 mg, molar ratio = 200:1), LiTFSI (added with the EO/Li^+^ = 12:1), DCP (3 wt% of the vinyl monomers), and EmimTFSI or Pyr_14_TFSI(ω = 0%, 26 wt%, 34 wt%, 42 wt%, and 50 wt%, where ω is the weight ratio of the composite polymer electrolyte) were dissolved in THF to form a homogeneous solution and mixed with precursor A. The solution was then casted onto a clean glass plate. After evaporation of the solvent at room temperature, the sample was first cured at 80 °C for 3 h and then polymerized at 115 °C for 24 h under nitrogen. The final obtained composite polymer electrolytes are denoted as CPE-E/P(ω), where E and P are EmimTFSI and Pyr_14_TFSI, respectively. Furthermore, a solid polymer electrolyte (SPE) without ionic liquid was prepared for comparison.

### 2.3. Measurements

Fourier transform infrared spectroscopy (FTIR) was measured by a German Brook spectral instrument VERTEX70, with a frequency range of 4000–600 cm^−1^. A differential scanning calorimetry (DSC, German Nechi 204F1) was used to test the phase behavior of the samples with a heating/cooling rate of 10 °C min^−1^. A thermogravimetric analysis test (TGA, NETZSCH TG 209F3) was performed from room temperature to 600 °C at a heating rate of 10 °C min^−1^ under a nitrogen atmosphere. Scanning electron microscopy (SEM) and energy-dispersive spectroscopy (EDS) (Nova NanoSEM 430, The Netherlands) were carried out to study the surface and cross-section morphologies of the CPEs. AC impedance spectroscopy measurements were performed with stainless steel blocking electrodes. The ionic conductivity (σ) in a temperature range of 18~100 °C is calculated with the following equation:

(1) $$\sigma =\frac{L}{R\;S}$$

Herein, *L*, *S,* and *R* are the thickness, surface area, and resistance of the sample. Linear scanning voltammetry (LSV) was measured to evaluate the electrochemical stability window (ESW) at a scan rate of 1.0 mV s^−1^, with stainless steel as the working electrode and lithium foil as the reference and counter electrodes. AC impedance spectroscopy measurements and chronoamperometry were used to study the lithium ion transference number [35,36]. The equation is as follows:

(2) $${t_{\mathrm{Li}}}^{+}=\frac{I_{s}}{I_{0}}\left(\frac{∆V-I_{0}R_{0}}{∆V-I_{s}R_{s}}\right)$$

where ∆V is the constant voltage applied. $I_{0}$ is the initial current, $\mathrm{and}\;I_{ss}$ is the steady-state current. $R_{0}$ and $R_{ss}$ are the interfacial resistance before and after DC polarization, respectively. The above electrochemical tests were carried out using a Metrohm electrochemical workstation (PGSTAT302N, Utrecht, The Netherlands).

Galvanostatic cycling tests of lithium symmetric cells were performed with 1 h charge/discharge cycling at different current densities. The thickness of the electrolyte film was about 150 μm to 200 μm. LiFePO_4_ electrodes were prepared in the same manner as in previous work [15]. The composition of active materials/binder/carbon black was 60/32/8, and the active material loading was about 3.0–3.5 mgΔcm^−2^. LiFePO_4_/CPE/Li cells were assembled by sandwiching the CPE between the LiFePO_4_ cathode and the lithium foil in an argon-filled glove box. The thickness of the composite polymer electrolyte was about 150 μm. The theoretical capacity of LiFePO_4_ is 170 mAh g^−1^, and the potential window is 2.5–4.0 V at 20 °C. The batteries were activated with a low current density before their further testing. All cells were 2032-type coin cells, and all cycling tests were carried out on the Land battery system (Wuhan LAND electronics Co., Ltd., Wuhan, China).

## 3. Results and Discussion

### 3.1. Preparation and Characterization of the Composite Polymer Electrolytes (CPEs)

The synthetic route of the double-network (DN) polymer electrolyte and CPEs is depicted in Scheme 1. A ring-opening reaction between the amino groups and the epoxy groups takes place rapidly at 80 °C to form a relatively hard network that has a high cross-linking density to provide mechanical support. Then, radical polymerization between poly(ethylene glycol) methyl ether acrylate (PEGMEA) and poly(ethylene glycol) diacrylate (PEGDA) is initiated by dicumyl peroxide (DCP) at 115 °C to obtain another network with a lower cross-linking density, where the short grafted PEG chains provide great mobility of the EO segments to increase the conductivity of lithium ions. The weight ratio of the two networks is 1:1. The lithium salt is bis(trifluoromethane)sulfonamide lithium salt (LiTFSI) with an EO:Li^+^ ratio of 16:1 in the CPEs. The ILs, 1-ethyl-3-methylimidazolium bis(trifluoromethylsulfonyl)imide (EmimTFSI) or 1-butyl-1-methylpyrrolidinium bis(trifluoromethylsulfonyl)imide (Pyr_14_TFSI), are dispersed in the polymer matrix and cooperate with EO segments to conduct lithium ions. In this work, the DN polymer electrolyte without any ionic liquid is denoted as SPE, and CPEs with EmimTFSI or Pyr_14_TFSI are denoted as CPE-E(ω) or CPE-P(ω), where ω is the weight percentage of IL in the CPEs.

**Figure 1:**
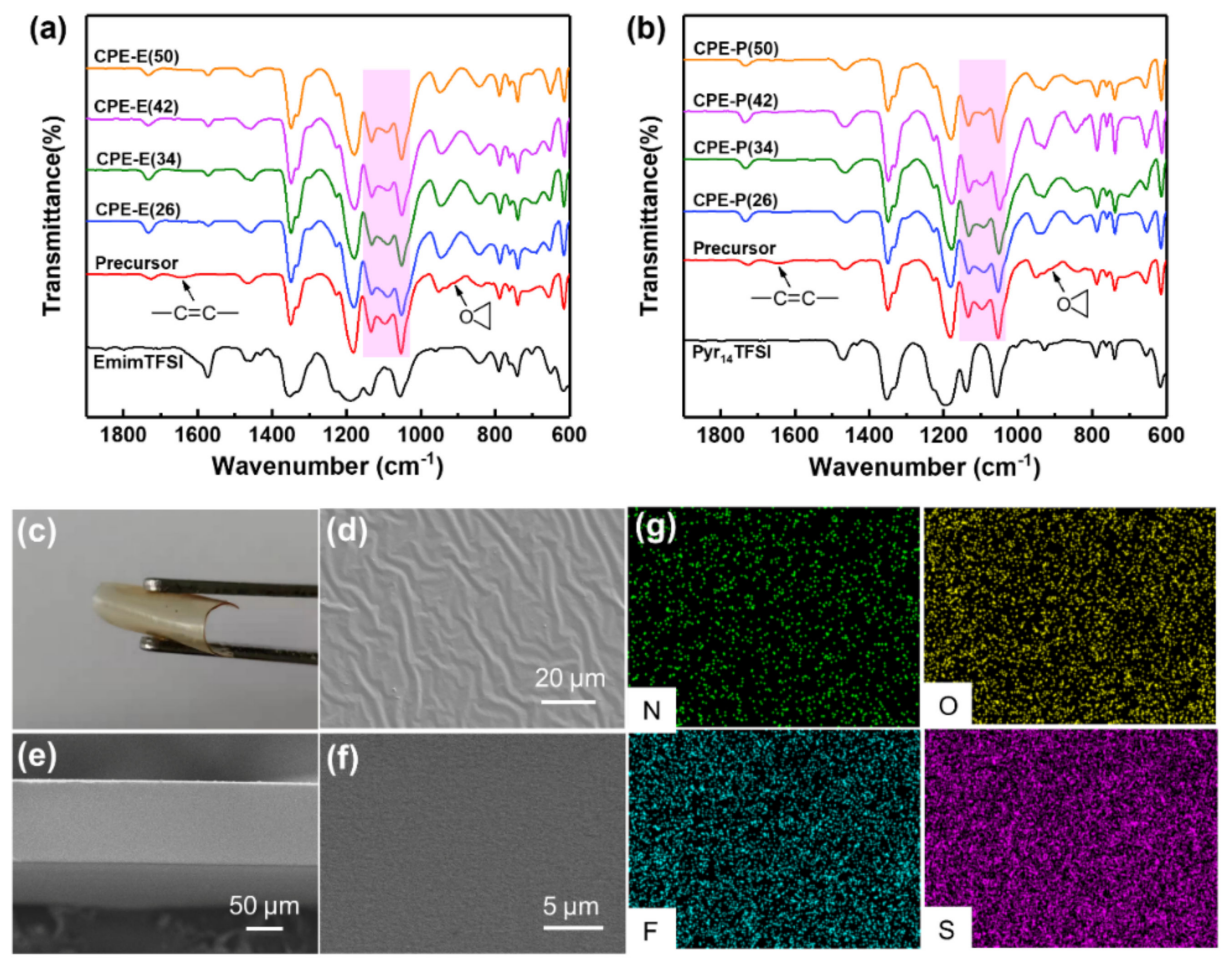
The FTIR spectra between 2000 and 600 cm^−1^ of the ionic liquids, precursors, and different CPEs (**a**,**b**). The photograph (**c**), surface SEM image (**d**), and cross-section SEM images (**e**,**f**) of the film of CPE-E(34). (**g**) EDS elemental mapping of the cross-section of the film of CPE-E(34).

Fourier transform infrared spectroscopy (FTIR) was used to characterize the structures of CPEs. As shown in Figure 1a,b, the characteristic bands of the precursor at 910 cm^−1^ and 1640 cm^−1^ correspond to the epoxy group and unsaturated -C=C-, respectively. They all disappear in the spectra of different CPE-E(ω) and CPE-P(ω), indicating the success of both the ring-opening reaction and free radical polymerization [15,37]. Moreover, the signal of C-O-C at 1100 cm^−1^ is weakened, and two new shoulder peaks appear at 1050 cm^−1^ and 1135 cm^−1^, which shows that lithium ions still form strong complexes with EO segments rather than forming ion pairs with ILs [38].

The free-standing and flexible CPE film can be seen in Figure 1c. The typical wrinkled structure of the cross-linked polymer is observed on the surface of the membrane under SEM (Figure 1d) [39]. Figure 1e,f are SEM images of the cross-section of the film of CPE-E(34), which show a smooth and compact surface without particle aggregation and pores. The corresponding elemental mapping of N, O, F, and S by energy-dispersive spectroscopy (EDS) (Figure 1g) also demonstrates the homogeneity of the prepared CPEs. Similar results are found for CPE-P(34) (Figure S1).

**Figure 2:**
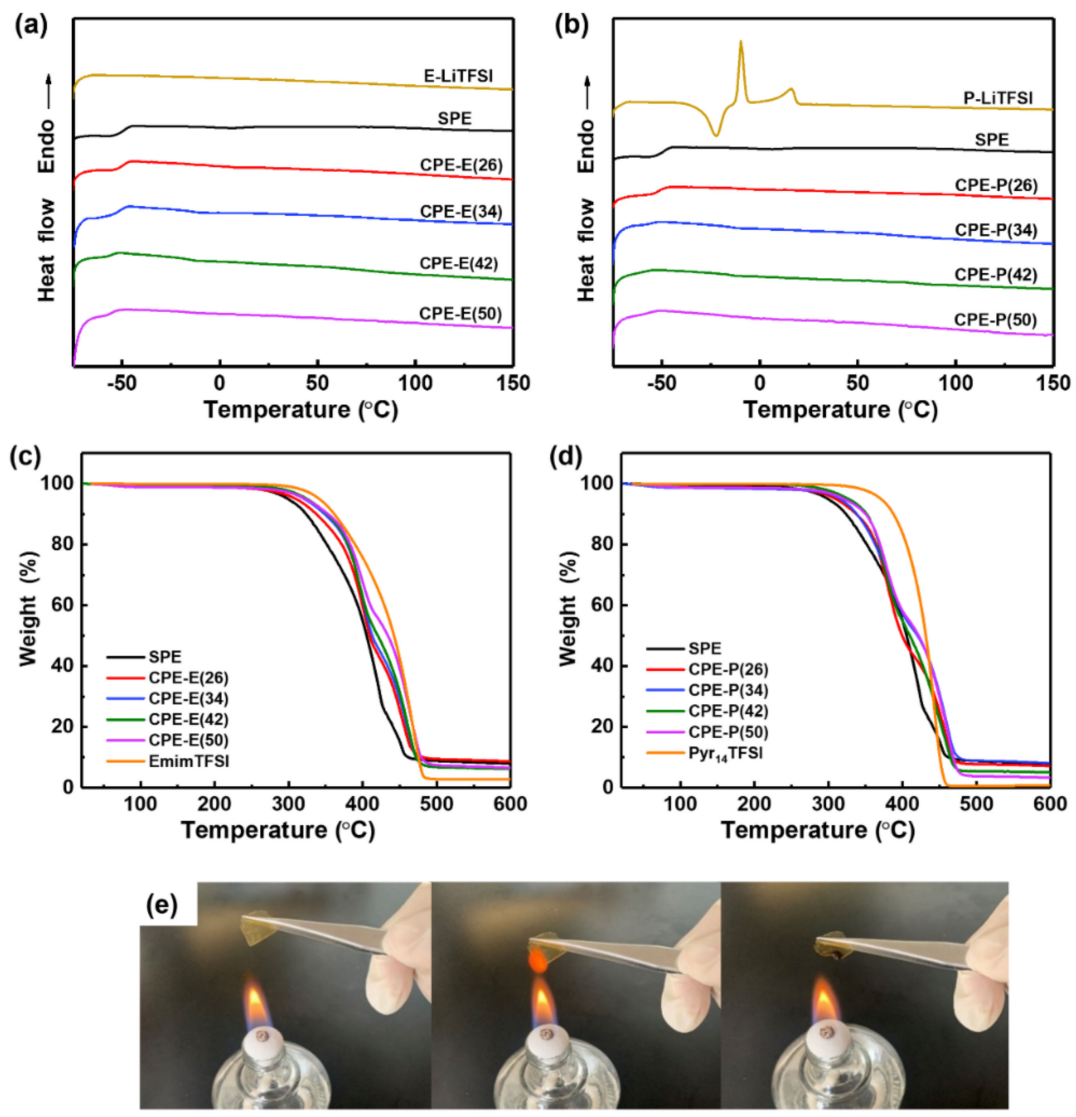
Thermal properties of E/P-LiTFSI solutions, SPE, and different CPEs: (**a**,**b**) DSC curves. (**c**,**d**) TGA curves. (**e**) Flammability test of CPE-E(34).

Differential scanning calorimetry (DSC) curves (Figure 2a,b) show the phase behaviors of IL-LiTFSI, SPE, and CPEs. In the results of the solution with 0.6 mol kg^−1^ LiTFSI in ionic liquids, denoted as E-Li and P-Li, respectively, there is no phase transition observed in E-Li, while P-Li shows obvious crystallization (−22.4 °C) and melting (−9.8 °C and 15.42 °C), which indicates that EmimTFSI has a lower melting point and better compatibility with lithium salts [34]. For SPE and all CPEs, only glass transitions (*T*_g_) are observed, suggesting that the ILs are well dispersed in the CPEs. At the same time, *T*_g_ decreases gradually with increasing amounts of ILs in the CPEs, and the values are shown in Table 1, which indicates that the ILs improve the segment mobility of the polymer matrix and enhance the migration of Li^+^, suggesting the high ionic conductivity of CPEs at room temperature [40].

Thermogravimetric analysis (TGA) was used (Figure 2c,d) to study the thermal stability of the SPE and CPEs. All CPEs show two-step decomposition before 500 °C, corresponding to the decomposition of the polymer network and ILs. The temperatures (*T*_d_) of 5% weight loss of the CPEs and SPE are also listed in Table 1. The SPE exhibits a *T*_d_ of 302 °C. Incorporation of the ILs and the DN polymer promotes the thermal stability of CPEs, which show a *T*_d_ above 316 °C. Meanwhile, the higher *T*_d_ values of CPE-P(ω) prove that Pyr_14_ TFSI has better thermal stability and safety, which corresponds to related reports [25,41].

The flammability test of CPE-E(34) is shown in Figure 2e. Only slight burning and shrinkage occurred when the sample was exposed to a flame for several seconds. Then, the flame went out automatically when the sample was moved away from the ignition source, confirming the low flammability and self-extinguishing property of the CPEs, which greatly improves the safety of lithium metal batteries (LMBs).

It is essential to reach a trade-off between the mechanical properties and electrochemical performance of CPEs because their mechanical strength decreases with increasing IL content. The mechanical properties of the CPEs were estimated by atomic force microscopy (AFM) force mapping, as shown in Figure S2. Both CPEs show good mechanical properties, which are ascribed to the DN structure, where the network with high cross-linking density provides mechanical support. The average Young’s modulus of the CPE-P(26) sample is higher than that of CPE-E(26).

### 3.2. Electrochemical Properties of the Composite Polymer Electrolytes (CPEs)

**Figure 3:**
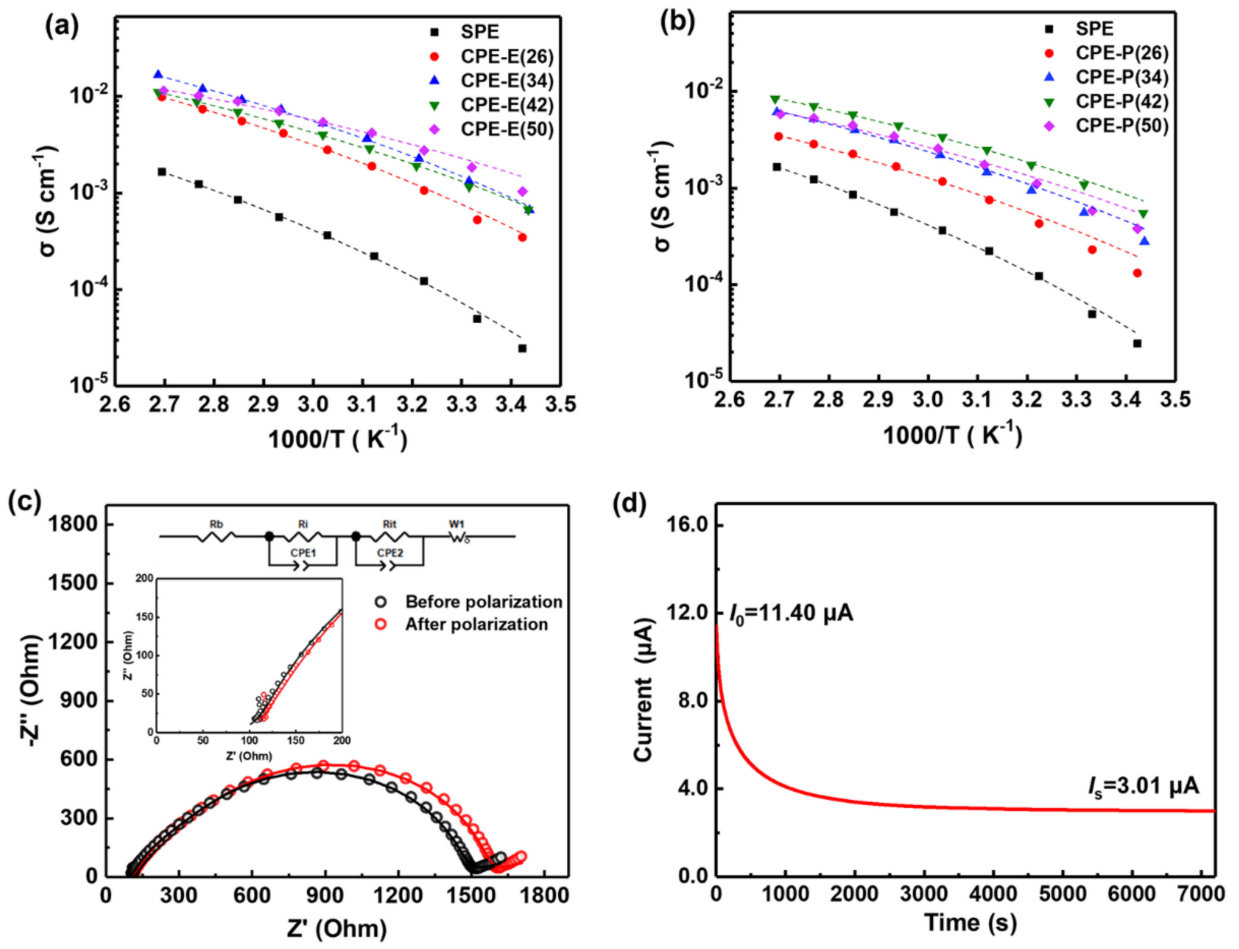
Temperature dependence of ionic conductivity for SPE and different CPEs (**a**,**b**). Nyquist plots before and after polarization (**c**) and the polarization curve (**d**) of the Li|CPE-P(34)|Li cell with a potential step of 20 mV at 20 °C.

High ionic conductivity (σ) is essential for LMBs to operate in a wide temperature range. The temperature-dependent ionic conductivity of different CPE samples is depicted in Figure 3a,b, and the σ values at 20 °C and 60 °C are listed in Table 1. Detailed information about the calculation of σ is shown in the Supporting Information (Figure S3). The solid-state SPE has an ionic conductivity of 2.6 × 10^−5^ S cm^−1^ at 20 °C, which is relatively high in the PEO-based SPE because of the high mobility of the short grafted PEG chains. To meet the need for batteries to run at room temperature or lower, EmimTFSI or Pyr_14_TFSI was added to the DN polymer. With a IL weight fraction of 26%, the ionic conductivities of both CPEs exceed 0.1 mS cm^−1^ at 20 °C: 0.41 mS cm^−1^ for CPE-E(26) and 0.15 mS cm^−1^ for CPE-P(26). The ionic conductivity of CPE-E(ω) is higher than that of CPE-P(ω) at the same IL content due to the lower viscosity and better compatibility with the lithium salt of EmimTFSI [42]. For CPEs with EmimTFSI, the ionic conductivity increases with increasing IL content, which exceeds 1 mS cm^−1^ for CPE-E(50) at 20 °C. However, for CPEs incorporated with Pyr_14_TFSI, the ionic conductivity first increases and then decreases with the increasing content of IL. CPE-P(42) exhibits the highest value, 0.6 mS cm^−1^ at 20 °C. The ionic conductivities of CPE-E(50) and CPE-P(42) exceed that of the CPE with a semi-interpenetrating network and a similar amount of the IL-based cocktail electrolyte [32]. Moreover, the ionic conductivity of CPE-P(42) is close to that of the gel polymer electrolyte (GPE) based on a single network and 60 wt% Pyr_13_TFSI [5]. We suppose that the dangling short EO chains in the DN contribute significantly to the high ionic conductivities of the CPEs here.

The ionic conductivities of the two types of CPEs show different trends with the content of ILs, indicating the different interactions between the lithium salt, ILs, and the polymer matrix. The ionic conductivities of the CPEs here are as complicated as some gel polymer electrolytes [43,44,45]. Both the PEO-based double network and the IL can dissolve the lithium salt, and the movement of the IL also contributes to the ionic conductivity. EmimTFSI is compatible with LiTFSI. Only a glass transition can be observed in the DSC experiment (Figure 2a). However, when mixing Pyr_14_TFSI with LiTFSI, the mixed salt crystalline phase is formed easily (Figure 2b). Crystallization can be detected when the content of LiTFSI varies from 0.05 to 0.75 [46,47]. It is speculated that when the IL is added to the double-network SPE, it mainly acts as a plasticizer at the beginning, which greatly increases the EO segment motion and thus increases the ionic conductivity. However, when Pyr_14_TFSI increases to a certain amount, it removes the lithium ions that complex with the EO segments, resulting in a decline in segment mobility (decreased *T*_g_, as shown in Table 1) and the formation of nano-crystals, leading to the decreasing ionic conductivity of CPE-P(50).

The lithium ion transference number ($t_{Li^{+}}$) is important for characterizing the effective conduction of lithium ions in CPEs. It was measured by the electrochemical method using a lithium symmetric cell (Figure 3c,d and Figure S4) [35,36]. The values were calculated by Equation (2) and are shown in Table 1. The $t_{Li^{+}}$ of the SPE is 0.14, and those of the two types of CPEs are in the range of 0.08–0.1. In the CPE, the addition of the IL introduces more anionic TFSI^−^, which inevitably leads to a decreased $t_{Li^{+}}$. The lowered values are also reported in other research [48,49]. It is worth noting that the$\;t_{Li^{+}}$ of CPE-E(ω) is slightly higher than that of CPE-P(ω) in most of the IL content owing to the higher dielectric constant of EmimTFSI, which helps to promote lithium salt dissociation and then to release more free Li^+^. Meanwhile, the cation Emim^+^ has stronger electrostatic interaction with the EO segment, which can weaken the complexation between Li^+^ and EO and accelerate lithium ion migration [50].

**Figure 4:**
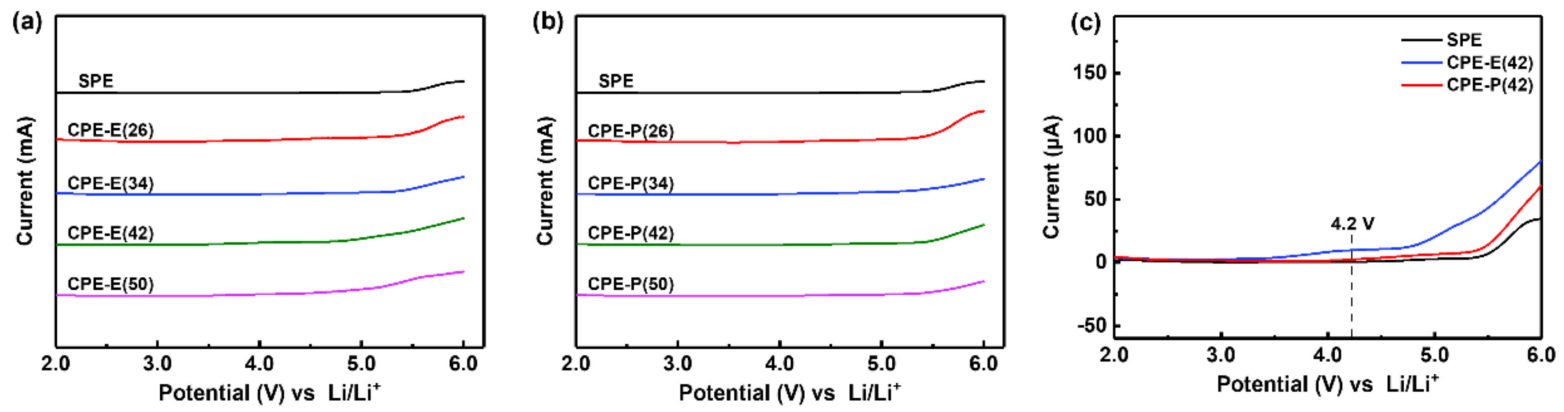
LSV curves of CPE-E(ω) (**a**) and CPE-P(ω) (**b**) at 25 °C, respectively. (**c**) Enlarged LSV curves for SPE, CPE-E(42) and CPE-P(42).

The electrochemical stability window (ESW) is another crucial factor for the application of electrolytes. As shown in Figure 4a,b, the linear sweep voltammetry (LSV) curves of the SPE and different CPEs exhibit large current responses after 5.0 V (vs. Li/Li^+^), corresponding to the decomposition of PEO chains. The LSV curves of the SPE, CPE-E(42), and CPE-P(42) are enlarged and shown in Figure 4c to further characterize the electrochemical stability of the two CPEs. The slight current response of CPE-E(42) before 4.0 V indicates that slight oxidation decomposition has already occurred. In contrast, CPE-P(42) exhibits similar electrochemical stability to the SPE owing to the electrochemical stability of Pyr_14_TFSI. On the other hand, it also indicates that the formation of the DN polymer by two-step polymerization is not affected when incorporating a higher content of Pyr_14_TFSI. In fact, the ESW around 4.0 V matches the potential window of the LiFePO_4_ cathode.

### 3.3. Study of the Resistance to Lithium Dendrite Growth of the CPEs

**Figure 5:**
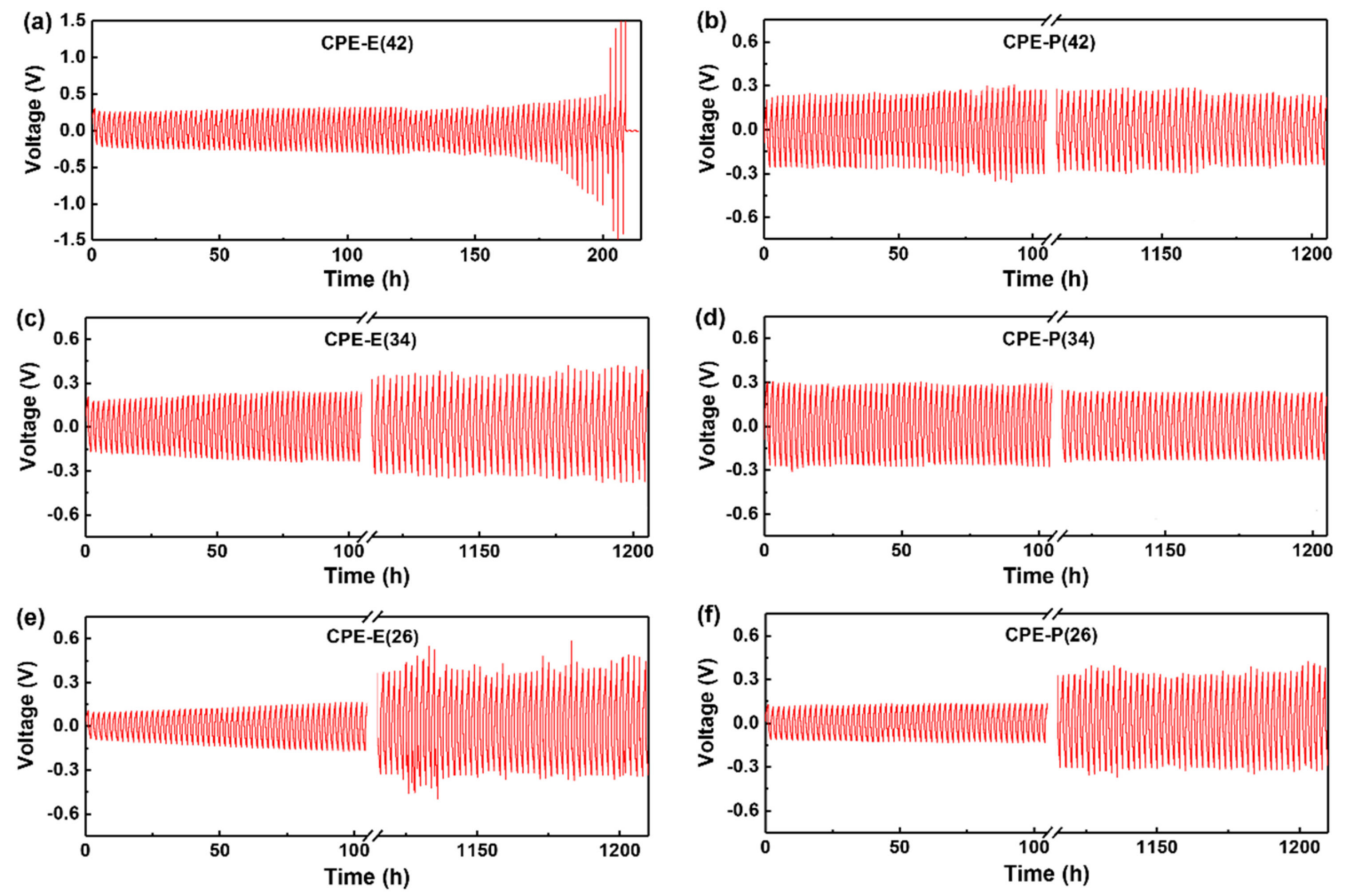
The voltage profiles of galvanostatic cycling of different lithium symmetric cells at a current density of 0.05 mA cm^−2^ at 20 °C: (**a**) Li|CPE-E(42)|Li, (**b**) Li|CPE-P(42)|Li, (**c**) Li|CPE-E(34)|Li and (**d**) Li|CPE-P(34)|Li cells at 20 °C, (**e**) Li|CPE-E(26)|Li, and (**f**) Li|CPE-P(26)|Li cells.

The growth of lithium dendrites is a great obstacle to the application of lithium metal anodes. Here, the CPE films were placed between two lithium foils to assemble lithium symmetric cells with 2032-type coil cells. The galvanostatic cycling test was then carried out to study the resistance to lithium dendrite growth of CPEs. The voltage profiles of the cells with different CPEs at 20 °C at a current density of 0.05 mA cm^−2^ are shown in Figure 5. Comparing the cycling performance of CPE-E(26) and CPE-P(26), although no sudden voltage decrease due to the short circuit of the cell is observed during the 1250 h cycling, the voltage of the cell with CPE-P(26) is more stable than that of the cell with CPE-E(26). With more Pyr_14_TFSI incorporated into the DN polymer, the potentials of the cells with CPE-P(34) and CPE-P(42) remain steady during the 1250 h cycling, indicating very stable CPE/Li metal interfaces during lithium deposition. For the cell with CPE-E(34), more even lithium deposition occurs than in the cell with CPE-E(26), which is attributed to the higher ionic conductivity of CPE-E(34). However, when increasing the weight fraction of EmimTFSI to 42%, short circuiting of the cell occurs at around 200 h. The possible reason is that with the increasing content of EmimTFSI, the mechanical properties of the CPE decrease rapidly, and the side reaction with lithium metal becomes more severe. The above results show that the CPEs with Pyr_14_TFSI form stable solid electrolyte interphase (SEI) layers on the surface of lithium metals, leading to stable galvanostatic cycling of the lithium symmetric cells, while in the CPEs with EmimTFSI, less stable lithium deposition occurs, where lithium dendrites short circuit the cell with a high content of IL.

To clearly evaluate the ability to inhibit the growth of lithium dendrites of the CPEs, galvanostatic polarization tests with lithium symmetric cells were performed at 20 °C at 0.03 mA cm^−2^. The obtained results are shown in Figure S5, where the sudden decrease in cell voltage to zero corresponds to the short circuiting of the cell by lithium dendrites. The short-circuit times of the cells with the two types of CPEs decrease gradually with increasing IL content. In addition, the short-circuit times of CPE-P(ω) are longer than those of CPE-E(ω), which reflects their increased resistance to the growth of lithium dendrites.

**Figure 6:**
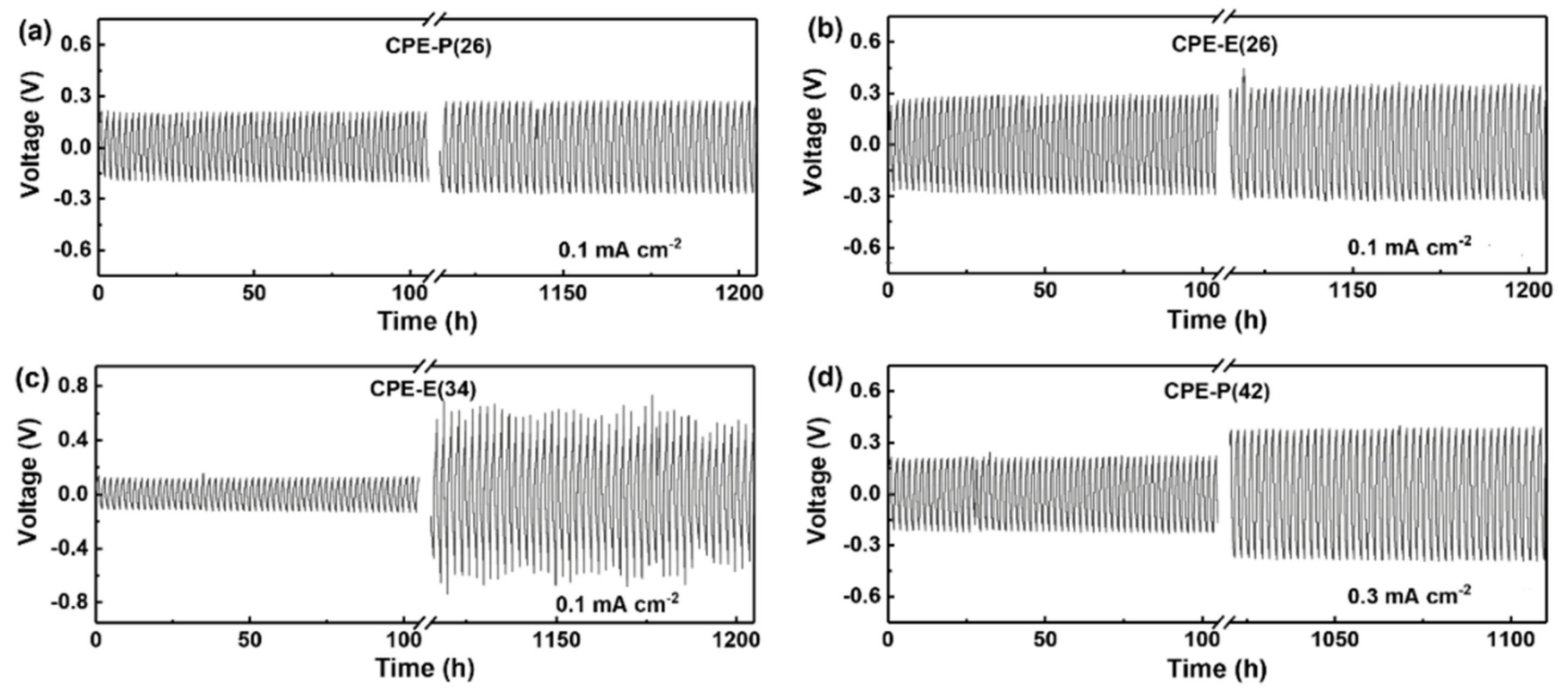
Voltage profiles of lithium symmetric cells for CPE-P(26) (**a**), CPE-E(26) (**b**), and CPE-E(34) (**c**) at 0.1 mA cm^−2^ and CPE-P(42) at 0.3 mA cm^−2^ (**d**) at 60 °C.

Furthermore, when the galvanostatic cycling tests of Li|CPEs|Li cells were performed at 60 °C, the cells with CPE-E(26) and CPE-P(26) all exhibited long and stable cycling at a current density of 0.1 mA cm^−2^ (Figure 6a,b). The cell with the CPE-P(42) sample shows excellent cycling stability with a current density of 0.3 mA cm^−2^ (Figure 6d). However, the voltage of the cell with CPE-E(34) greatly increases and becomes unstable during long-term cycling, indicating serious side reactions on the CPE/Li interface at an elevated temperature. Comparing the above results, the CPE-P(ω) samples possess a long cycling life of over 1000 h and stable cell voltages at both 20 °C and 60 °C, suggesting their excellent resistance to lithium dendrite growth. This is attributed to the excellent electrochemical stability of Pyr_14_TFSI, the stable SEI layer, and the better mechanical strength of CPE-P(ω) [42,51].

**Figure 7:**
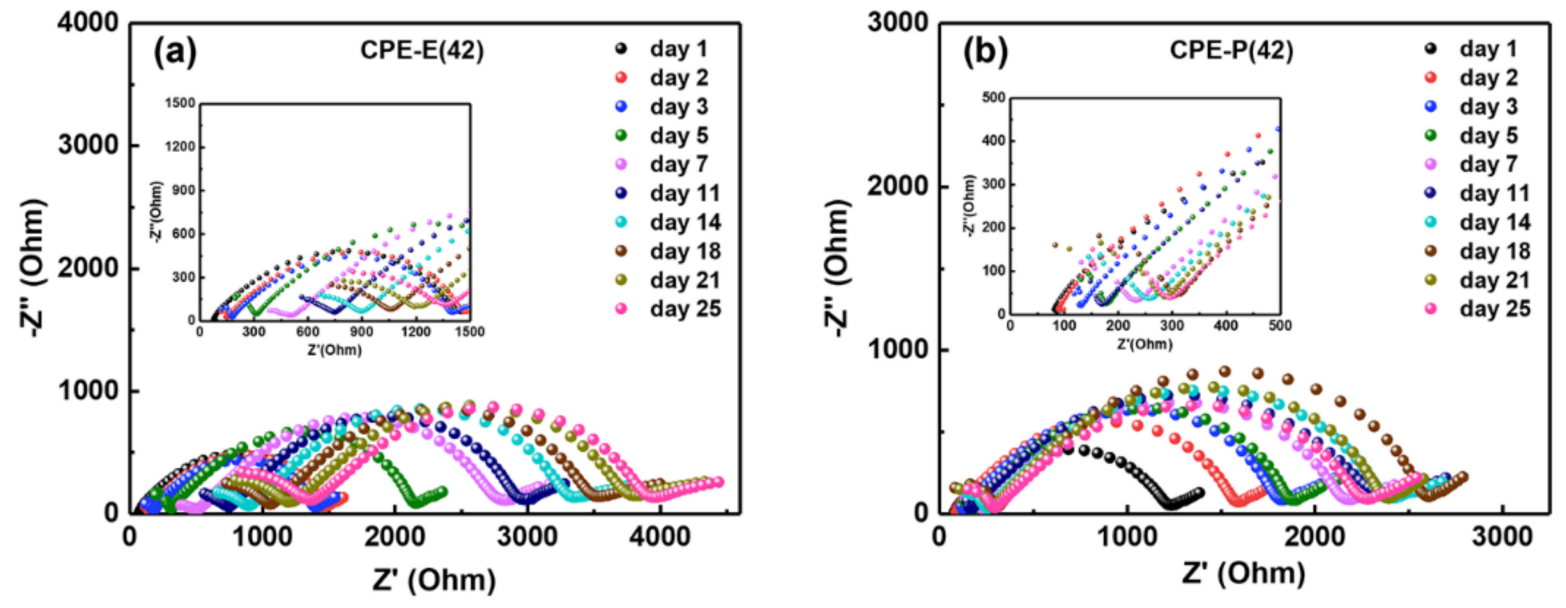
Nyquist plots of Li|CPE-E(42)|Li (**a**) and Li|CPE-P(42)|Li (**b**) cells over time at room temperature.

In addition, cells of Li|CPE-E(42)|Li and Li|CPE-P(42)|Li were assembled and stored at room temperature to further characterize the stability of high-IL-content CPEs to lithium metal. In Nyquist plots of symmetric cells after various storage times (Figure 7), the bulk and interface resistances of the cell with CPE-P(42) increase at first, but after 18 days, the interface resistance slightly decreases, without any increasing trend during the next week. However, the interface resistances of CPE-E(42) increase gradually over time without a steady trend. Therefore, the CPEs with Pyr_14_TFSI are more stable to the lithium anode than those with EmimTFSI, which is essential for the storage and application of LMBs.

### 3.4. Performance of Li/LiFePO_4_ Batteries

**Figure 8:**
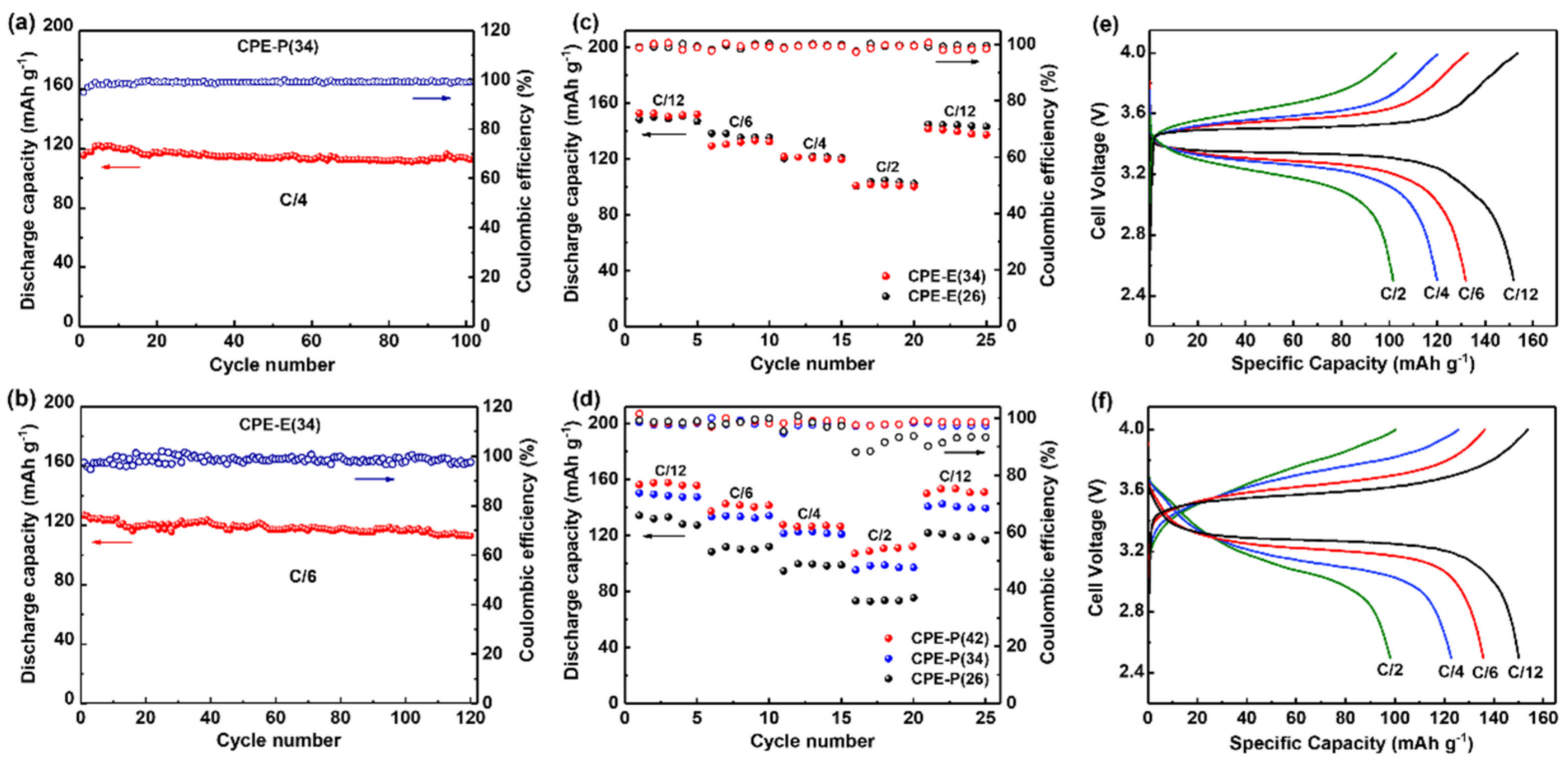
Cycling stability and coulombic efficiency of Li/LiFePO_4_ cells with CPE-P(34) at a C/4 rate (**a**) and CPE-E(34) at a C/6 rate (**b**) at 20 °C. Rate performance and corresponding coulombic efficiency of Li/LiFePO_4_ cells with CPE-E (**c**) and CPE-P (**d**) at 20 °C. Voltage profiles for the cells of Li|CPE-E(34)|LiFePO_4_ (**e**) and Li|CPE-P(34)|LiFePO_4_ (**f**) at different current rates at 20 °C.

It is important to meet the practical application requirements of LMBs at room temperature. Based on the excellent ionic conductivities of the CPEs, and considering the cost of ILs, CPE-E(34) and CPE-P(34) were selected as solid-state electrolytes to assemble Li/LiFePO_4_ batteries. The corresponding galvanostatic cycling tests were then carried out at 20 °C. The cell with CPE-P(34) delivers the highest discharge capacity of 124 mAh g^−1^ at a C/4 rate and shows good cycling stability with a 96% capacity retention after 100 cycles. The average coulombic efficiency for the cell is 98.8% (Figure 8a). By contrast, the cycling stability and coulombic efficiency of the Li|CPE-E(34)|LiFePO_4_ cell are inferior to those of the CPE-P(34)-based one, although CPE-E(34) has higher ionic conductivity. The original capacity of the cell is 128 mAh g^−1^ at a C/6 rate with an average coulombic efficiency of 98.0% (Figure 8b). A faster capacity attenuation is observed after 100 cycles, where 8% capacity decay is observed. The above results indicate the electrochemical stability of CPE-P(ω) and the favorable reversible Li^+^ extraction/insertion behavior during cycling, suggesting the unique performance of the Pyr_14_TFSI ionic liquid [52,53]. CPE-P(34) shows better resistance to the growth of lithium dendrites than CPE-E(34), indicating a more stable SEI layer between CPE-P(34) and the lithium metal. This stable SEI layer is the main reason for the better discharge/charge efficiency and stability in the corresponding LFP cell.

The rate capability of Li/LiFePO_4_ cells with different CPEs at different current rates and 20 °C was carefully studied. Cells were continuously charged and discharged for five cycles at different current densities, from C/12 to C/2 and then back to C/12. The results are shown in Figure 8c,d. For all cells, the stable discharge capacities are observed at each discharge rate, although the capacities decrease with increasing current densities, which recover to the initial values at C/12 after cycling at higher rates. The discharge capacities of the cell with CPE-P(42) are higher than those with CPE-P(34) and CPE-P(26) at different current rates, which is ascribed to the high ionic conductivity, while there is no significant difference in the discharge capacity of cells with CPE-E(26) and CPE-E(34), which can be explained by the similar interface properties and sufficiently high ionic conductivities. Comparing the cells with CPE-P(26) and CPE-E(26), the latter one has the better rate capability, where capacities of 137 mAh g^−1^ vs. 145 mAh g^−1^ at a C/12 rate and 75 mAh g^−1^ vs. 102 mAh g^−1^ at a C/2 rate are observed. This is attributed to the much lower ionic conductivity of CPE-P(26) compared to that of CPE-E(26). For cells with CPE-P(34) and CPE-E(34), the rate capabilities are similar, where a capacity of 152 mAh g^−1^ at a C/12 rate and a capacity of 102 mAh g^−1^ at a C/2 rate are achieved. The cell with CPE-P(42) shows the best rate capacity among all LMBs. The discharge capacities are 157, 143, 128, and 118 mAh g^−1^ at C/12, C/6, C/4, and C/2. Although the IL dosage of CPE-P(42) is much lower than that of the GPE composed of a single PEO network and 83 wt% Pyr_13_TFSI, its cell shows better rate capability, which shows the advantage of the DN CPEs in this work [5].

The corresponding voltage profiles of Li|CPE-E(34)|LiFePO_4_ and Li|CPE-P(34)| LiFePO_4_ cells are depicted in Figure 8e,f, respectively. Well-defined potential plateaus are observed up to a C/2 rate for all cells. The CPE-P(34)-based cell shows larger overpotentials and lower discharge potential plateaus than those of CEP-E(34) at the same current rate, which is ascribed to its higher bulk resistance. The declining discharge plateau potentials observed when increasing the current rate are a result of increased polarization potentials at a high current rate [54].

## 4. Conclusions

To meet the application requirements of solid-state electrolytes in lithium metal batteries at room temperature, two kinds of ionic liquids (ILs), EmimTFSI or Pyr_14_TFSI, were introduced to double-network (DN) polymer electrolytes by the one-pot synthetic route to obtain composite polymer electrolytes (CPEs). Benefitting from the DN polymer structure, where the short dangling EO chains are favorable for lithium transportation, 26 wt% ILs can increase the ionic conductivities of the CPEs to exceed 10^−4^ S cm^−1^ at 20 °C. The CPEs also show excellent thermal stability and a self-extinguishing property. Meanwhile, due to the different physical and chemical properties of the two ILs, the differences in the corresponding CPEs are highlighted. The CPEs with EmimTFSI show higher ionic conductivities than those with Pyr_14_TFSI at the same IL content. However, the CPEs with Pyr_14_TFSI exhibit a wider electrochemical stability window, better mechanical properties, and better resistance to the growth of lithium dendrites than the CPE-E samples. Although CPE-P(34) exhibits lower conductivity than CPE-E(34), their Li/LiFePO_4_ batteries exhibit similar rate capabilities, which deliver stable discharge capacities of 152 and 102 mAh g^−1^ at current rates of C/12 and C/2 at 20 °C, respectively. Moreover, the former shows better cycling stability and higher coulombic efficiency. According to the comprehensive results of this work, DN CPEs incorporating Pyr_14_TFSI are more promising candidates for next-generation solid-state LMBs.

## Figures and Tables

**Table 1:** Thermal properties, ionic conductivity (σ), and lithium ion transference number ($t_{Li^{+}}$ ) of different CPEs.

Samples	*T*_g_ (°C)	*T*_d_ (°C)	σ (20 °C, mS cm^−1^)	σ (60 °C, mS cm^−1^)	$t_{Li^{+}}$
SPE	−46.0	302	0.026	0.40	0.14
CPE-E(26)	−49.3	316	0.41	3.12	0.092
CPE-E(34)	−50.9	326	0.83	5.50	0.082
CPE-E(42)	−54.7	328	0.80	4.18	0.084
CPE-E(50)	−55.1	330	1.06	5.60	0.080
CPE-P(26)	−51.3	317	0.15	1.26	0.088
CPE-P(34)	−55.2	327	0.29	2.36	0.079
CPE-P(42)	−58.5	328	0.60	3.61	0.082
CPE-P(50)	−57.4	330	0.39	2.63	0.10

## Data Availability

The data presented in this study are available in this study and the corresponding Supplementary Materials.

## Supplementary Materials

The following supporting information can be downloaded at: https://www.mdpi.com/article/10.3390/polym14173435/s1. Figure S1: SEM images, Figure S2: Mechanical properties, Figure S3: Nyquist plot, Figure S4: Lithium transference number measurement, Figure S5: Galvanostatic polarization curves.

## References

[1] Xu W., Wang J.L., Ding F., Chen X.L., Nasybutin E., Zhang Y.H., Zhang J.G. (2014). Lithium metal anodes for rechargeable batteries. Energy Environ. Sci..

[2] Lu Y.X., Rong X.H., Hu Y.S., Chen L.Q., Li H. (2019). Research and development of advanced battery materials in China. Energy Storage Mater..

[3] Cheng X.B., Zhang R., Zhao C.Z., Wei F., Zhang J.G., Zhang Q. (2016). A review of solid electrolyte interphases on lithium metal anode. Adv. Sci..

[4] Vishnugopi B.S., Verma A., Mukherjee P.P. (2020). Morphology-safety implications of interfacial evolution in lithium metal anodes. J. Phys. Chem. C.

[5] Li X., Zheng Y., Li C.Y. (2020). Dendrite-free, wide temperature range lithium metal batteries enabled by hybrid network ionic liquids. Energy Storage Mater..

[6] Zhang X.Q., Zhao C.Z., Huang J.Q., Zhang Q. (2018). Recent advances in energy chemical engineering of next-generation lithium batteries. Engineering.

[7] Tikekar M.D., Choudhury S., Tu Z.Y., Archer L.A. (2016). Design principles for electrolytes and interfaces for stable lithium-metal batteries. Nat. Energy.

[8] Barai P., Higa K., Srinivasan V. (2017). Lithium dendrite growth mechanisms in polymer electrolytes and prevention strategies. Phys. Chem. Chem. Phys..

[9] Zhao W.J., Yi J., He P., Zhou H.S. (2019). Solid-state electrolytes for lithium-ion batteries: Fundamentals, challenges and perspectives. Electrochem. Energy Rev..

[10] Zhao S.S., Wu Q.X., Ma W.Q., Yang L.S. (2020). Polyethylene oxide-Based composites as solid-state polymer electrolytes for lithium metal batteries: A mini review. Front. Chem..

[11] Wang Q., Wang H.C., Wu J.Y., Zhou M.Y., Liu W., Zhou H.H. (2021). Advanced electrolyte design for stable lithium metal anode: From liquid to solid. Nano Energy.

[12] Zhou D., Shanmukaraj D., Tkacheva A., Armand M., Wang G.X. (2019). Polymer electrolytes for lithium-based batteries: Advances and prospects. Chem.

[13] Zhang X.W., Daigle J.C., Zaghib K. (2020). Comprehensive review of polymer architecture for all-solid-state lithium rechargeable batteries. Materials.

[14] Xu Z., Chu X., Wang Y.H., Zhang H.T., Yang W.Q. (2020). Three-dimensional polymer networks for solid-state electrochemical energy storage. Chem. Eng. J..

[15] Pan Q.W., Smith D.M., Qi H., Wang S.J., Li C.Y. (2015). Hybrid electrolytes with controlled network structures for lithium metal batteries. Adv. Mater..

[16] Choudhury S. (2019). A Highly Reversible Toom-Temperature Lithium Metal Battery Based on Cross-Linked Hairy Nanoparticles.

[17] Zhang Y., Lu W., Cong L., Liu J., Sun L., Mauger A., Julien C.M., Xie H., Liu J. (2019). Cross-linking network based on poly(ethylene oxide): Solid polymer electrolyte for room temperature lithium battery. J. Power Sources.

[18] Moreno J.S., Jeremias S., Moretti A., Panero S., Passerini S., Scrosati B., Appetecchi G.B. (2015). Ionic liquid mixtures with tunable physicochemical properties. Electrochim. Acta.

[19] Lewandowski A., Swiderska-Mocek A. (2009). Ionic liquids as electrolytes for Li-ion batteries-An overview of electrochemical studies. J. Power Sources.

[20] Osada I., de Vries H., Scrosati B., Passerini S. (2016). Ionic-liquid-based polymer electrolytes for battery applications. Angew. Chem. Int. Edit.

[21] Lee S., Park K., Koo B., Park C., Jang M., Lee H., Lee H. (2020). Safe, Stable cycling of lithium metal batteries with low-viscosity, fire-retardant locally concentrated ionic liquid electrolytes. Adv. Funct. Mater..

[22] Huo H., Zhao N., Sun J., Du F., Li Y., Guo X. (2017). Composite electrolytes of polyethylene oxides/garnets interfacially wetted by ionic liquid for room-temperature solid-state lithium battery. J. Power Sources.

[23] Zhou Y.D., Wang X.E., Zhu H.J., Armand M., Forsyth M., Greene G.W., Pringle J.M., Howlett P.C. (2018). Ternary lithium-salt organic ionic plastic crystal polymer composite electrolytes for high voltage, all-solid-state batteries. Energy Storage Mater..

[24] Cheng X.B., Hou T.Z., Zhang R., Peng H.J., Zhao C.Z., Huang J.Q., Zhang Q. (2016). Dendrite-free lithium deposition induced by uniformly distributed lithium ions for efficient lithium metal batteries. Adv. Mater..

[25] Gonzalez F., Tiemblo P., Garcia N., Garcia-Calvo O., Fedeli E., Kvasha A., Urdampilleta I. (2018). High performance polymer/ionic liquid thermoplastic solid electrolyte prepared by solvent free processing for solid state lithium metal batteries. Membranes.

[26] Karuppasamy K., Rhee H.W., Reddy P.A., Gupta D., Mitu L., Polu A.R., Shajan X.S. (2016). Ionic liquid incorporated nanocomposite polymer electrolytes for rechargeable lithium ion battery: A way to achieve improved electrochemical and interfacial properties. J. Ind. Eng. Chem..

[27] Xie Z.K., Wu Z.J., An X.W., Yoshida A., Wang Z.D., Hao X.G., Abudula A., Guan G.Q. (2019). Bifunctional ionic liquid and conducting ceramic co-assisted solid polymer electrolyte membrane for quasi-solid-state lithium metal batteries. J. Membrane Sci..

[28] Zhang M.Y., Becking J., Stan M.C., Lenoch A., Bieker P., Kolek M., Winter M. (2020). Wetting phenomena and their effect on the electrochemical performance of surface-tailored lithium metal electrodes in contact with cross-linked polymeric electrolytes. Angew. Chem. Int. Edit..

[29] Shen C., Huang Y.B., Yang J.R., Chen M.J., Liu Z.P. (2021). Unraveling the mechanism of ion and electron migration in composite solid-state electrolyte using conductive atomic force microscopy. Energy Storage Mater..

[30] Pan Q., Barbash D., Smith D.K., Qi H., Gleeson S.E., Li C.Y. (2017). Correlating electrode–electrolyte interface and battery performance in hybrid solid polymer electrolyte-based lithium metal batteries. Adv. Energy Mater..

[31] Nair J.R., Destro M., Bella F., Appetecchi G.B., Gerbaldi C. (2016). Thermally cured semi-interpenetrating electrolyte networks (s-IPN) for safe and aging-resistant secondary lithium polymer batteries. J. Power Sources.

[32] Nair J.R., Colo F., Kazzazi A., Moreno M., Bresser D., Lin R.Y., Bella F., Meligrana G., Fantini S., Simonetti E. (2019). Room temperature ionic liquid (RTIL)-based electrolyte cocktails for safe, high working potential Li-based polymer batteries. J. Power Sources.

[33] Gong J.P., Katsuyama Y., Kurokawa T., Osada Y. (2003). Double-network hydrogels with extremely high mechanical strength. Adv. Mater..

[34] Gong J.P. (2010). Why are double network hydrogels so tough?. Soft Matter.

[35] Bruce P.G., Evans J., Vincent C.A. (1988). Conductivity and transference number measurements on polymer electrolytes. Solid State Ion..

[36] Appetecchi G.B., Dautzenberg G., Scrosati B. (1996). A new class of advanced polymer electrolytes and their relevance in plastic-like, rechargeable lithium batteries. J. Electrochem. Soc..

[37] Fan W., Li N.W., Zhang X.L., Zhao S.Y., Cao R., Yin Y.Y., Xing Y., Wang J.N., Guo Y.G., Li C.J. (2018). A dual-salt gel polymer electrolyte with 3D cross-linked polymer network for dendrite-free lithium metal batteries. Adv. Sci..

[38] Vijayakumar V., Diddens D., Heuer A., Kurungot S., Winter M., Nair J.R. (2020). Dioxolanone-anchored poly(allyl ether)-based cross-linked dual-salt polymer electrolytes for high-voltage lithium metal batteries. Acs Appl. Mater. Inter..

[39] Lim J.Y., Kang D.A., Kim N.U., Lee J.M., Kim J.H. (2019). Bicontinuously crosslinked polymer electrolyte membranes with high ion conductivity and mechanical strength. J. Membrane Sci..

[40] Yang G., Song Y.D., Wang Q., Zhang L.B., Deng L.J. (2020). Review of ionic liquids containing, polymer/inorganic hybrid electrolytes for lithium metal batteries. Mater. Design.

[41] Kale S.B., Nirmale T.C., Khupse N.D., Kale B.B., Kulkarni M.V., Pavitran S., Gosavi S.W. (2021). Cellulose-derived flame-retardant solid polymer electrolyte for lithium-ion batteries. Acs Sustain. Chem. Eng..

[42] Appetecchi G.B., Montanino M., Carewska M., Moreno M., Alessandrini F., Passerini S. (2011). Chemical-physical properties of bis(perfluoroalkylsulfonyl)imide-based ionic liquids. Electrochim. Acta.

[43] Qian J., Jin B.Y., Li Y.Y., Zhan X.L., Hou Y., Zhang Q.H. (2021). Research progress on gel polymer electrolytes for lithium-sulfur batteries. J. Energy Chem..

[44] Chiu L.-L., Chung S.-H. (2021). A poly(ethylene oxide)/lithium bis(trifluoromethanesulfonyl)imide-loated polypropylene membrane for a high-loading lithium-sulfur battery. Polymers.

[45] Chiu L.-L., Chung S.-H. (2022). Composite gel-polymer electrolyte for high-loading polysulfide cathodes. J. Mater. Chem. A.

[46] Henderson W.A., Passerini S. (2004). Phase behavior of ionic liquid-LiX mixtures: Pyrrolidinium cations and TFSI- anions. Chem. Mater..

[47] Nadherna M., Dominko R., Hanzel D., Reiter J., Gaberscek M. (2009). Electrochemical behavior of Li_2_FeSiO_4_ with ionic liquids at elevated temperature. J. Electrochem. Soc..

[48] Wohde F., Balabajew M., Roling B. (2016). Li+ transference numbers in liquid electrolytes obtained by very-low-frequency impedance spectroscopy at variable electrode distances. J. Electrochem. Soc..

[49] Lassegues J.C., Grondin J., Talaga D. (2006). Lithium solvation in bis(trifluoromethanesulfonyl)imide-based ionic liquids. Phy. Chem. Chem. Phy..

[50] Kobayashi K., Pagot G., Vezzu K., Bertasi F., Di Noto V., Tominaga Y. (2021). Effect of plasticizer on the ion-conductive and dielectric behavior of poly(ethylene carbonate)-based Li electrolytes. Polym. J..

[51] Choi H., Kim H.W., Kim J.K., Lim Y.J., Kim Y., Ahn J.H. (2017). Nanocomposite quasi-solid-state electrolyte for high-safety lithium batteries. Nano Res..

[52] Li X.W., Zhang Z.X., Li S.J., Yang L., Hirano S. (2016). Polymeric ionic liquid-plastic crystal composite electrolytes for lithium ion batteries. J. Power Sources.

[53] Shin J.H., Henderson W.A., Tizzani C., Passerini S., Jeong S.S., Kim K.W. (2006). Characterization of solvent-free polymer electrolytes consisting of ternary PEO-LiTFSI-PYR14 TFSI. J. Electrochem. Soc..

[54] Li X.W., Zhang Z.X., Li S.J., Yang K.H., Yang L. (2017). Polymeric ionic liquid-ionic plastic crystal all-solid-state electrolytes for wide operating temperature range lithium metal batteries. J. Mater. Chem. A.

