# Supplementary material for: Double-Network Polymer Electrolytes with Ionic Liquids for Lithium Metal Batteries
Source: Polymers (Basel). 2022 Aug 23;14(17):3435. doi: 10.3390/polym14173435 (PMC9460741; doi:10.3390/polym14173435)
Supplement: Supplementary file 1 [file polymers-14-03435-s001.zip › polymers-1848339-supplementary.pdf]

polymers

MDPI

Supplementary Materials

Double-Network Polymer Electrolytes with Ionic Liquids for Lithium Metal Batteries

Chenjing Zhu [1], Yi Ning [1], Yizhi Jiang [1], Guangji Li [1] and Qiwei Pan [1,2,3,*]

[1] School of Materials Science and Engineering, South China University of Technology, Guangzhou 510640, China
[2] Key Laboratory of Polymer Processing Engineering, South China University of Technology, Ministry of Education, Guangzhou 510640, China
[3] Guangdong Provincial Key Enterprise Laboratory of Novel Polyamide 6 Functional Fiber Materials Research and Application, Jiangmen 529100, China
* Correspondence: panqw@scut.edu.cn

1. Polymerization efficiency

To evaluate the polymerization efficiency of the double network, the leaching tests are carried out. The film of the CPE was weighed before soaking in THF for 6 hours three times to remove the ionic liquid, LiTFSI, oligomers, and monomers. It was dried in an vacuum oven at 60 °C for 2 hours to remove the residual THF. The film was weighed again. The polymerization efficiency (ϕ) was calculated as following equation:

$$\phi = \frac{M_2}{xM_1} \times 100\% \quad (1)$$

Here, M_1 and M_2 are the weight of the polymer film before and after the extraction, respectively. x is the weight percentage of the double network in the CPE. At least three tests were conducted and the average number was taken as the final result. The ϕ of the SPE, CPE-E(26), CPE-P(26), CPE-E(50), and CPE-P(50) are 84.2%, 80.2%, 79.7%, 78.8%, and 78.5%, respectively. In summary, the ϕ of different CPE-P/E samples are quite close to the value of the SPE, which indicates the effective polymerization when containing large amounts of ionic liquid.

2. SEM images

The surface SEM image (a), and the cross-section SEM images (b) of CPE-P(34) sample are shown in Figure S1. The same wrinkled structure and compact morphology indicate the desirable cross-linked network of CPE-P(34). EDS results show that the element C, O, and F are evenly distributed in the CPE.

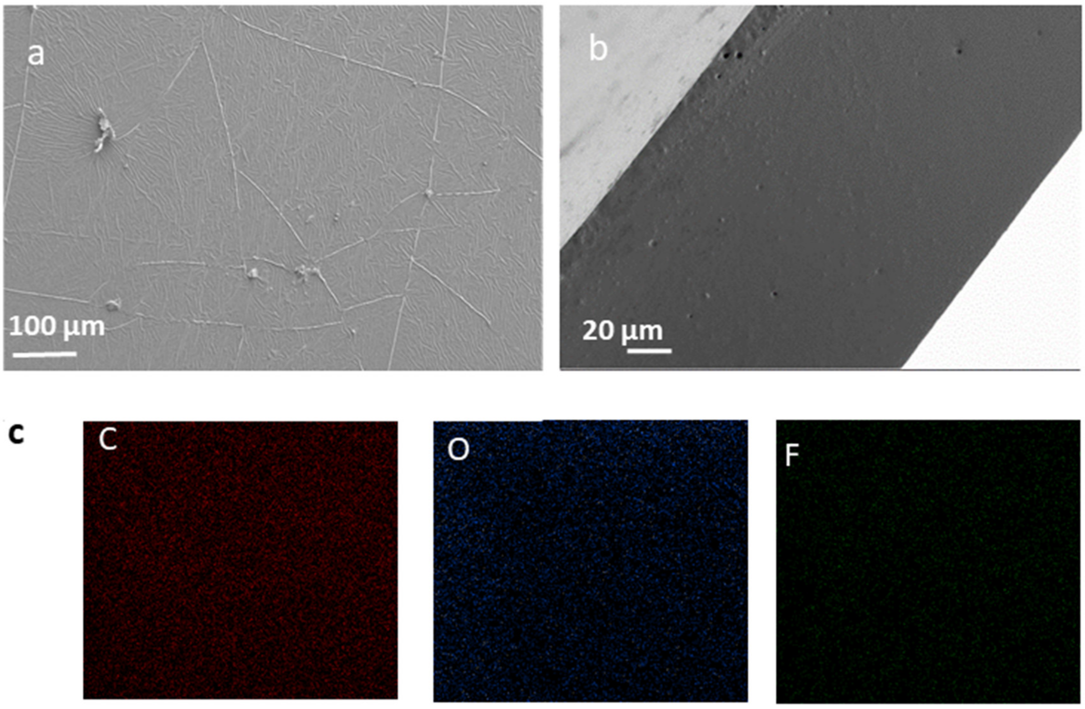

Figure S1. The surface SEM image (a), the cross-section SEM images (b), and the EDS elements mapping of the CPE-P(34) sample (c).

3. Mechanical Properties

In order to analyze the mechanical properties of the CPEs, CPE-E(26) and CPE-P(26) are characterized by AFM force mapping. The AFM experiments were conducted on a Bruker Multimode 8 AFM and a Bruker Dimension. The test results are shown in Figure S2. In the Young's modulus diagram, the mechanical strength of the two samples shows a wide distribution range, which may be due to the rough surface of the films. The different force curves of the two samples show a relatively smooth trend, indicating that the systems present relatively uniform structures distribution at a certain thickness. In addition, according to software analysis, the average mechanical strength of CPE-P(26) sample is higher than that of CPE-E(26), and its force curve also shows a higher force response (Force), indicating that CPE with Pyr14TFSI has higher mechanical properties, which may be attributed to the higher viscosity of Pyr14TFSI. It can be inferred that the mechanical modulus of CPE-E(ω) and CPE-P(ω) systems will decrease as the proportion of polymer phase decreases when increasing the IL content, but the mechanical strength of CPE-P(ω) systems should be higher with the same ionic liquid content.

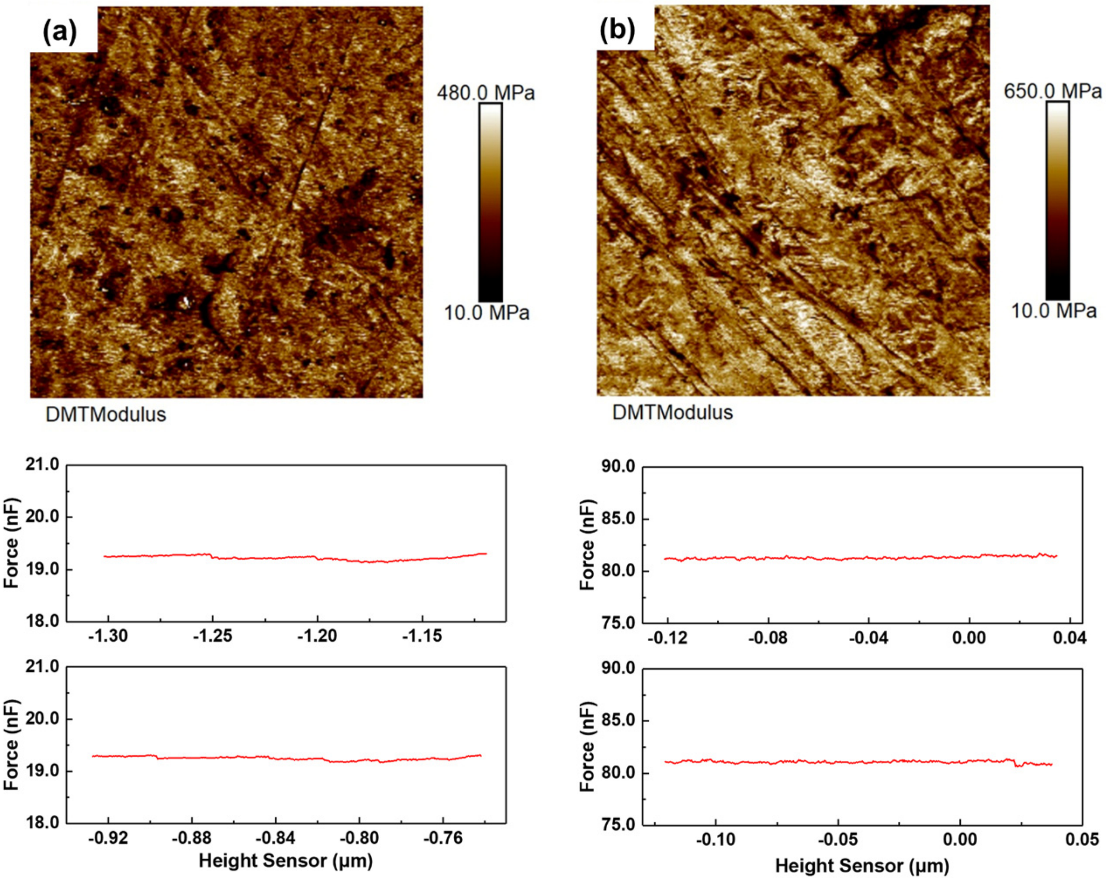

Figure S2. Young's modulus diagrams and force curves of (a) CPE-E(26) and (b) CPE-P(26) samples measured by AFM.

4. Ionic conductivity

The ionic conductivities(σ) of different CPE-P/E samples were calculated by equation

$$\sigma = \frac{L}{R\,S} \tag{2}$$

Here, *L*, *S*, and *R* are the thickness, surface area, and resistance of the sample. Taking the CPE-P(50) for example, the CPE film was placed between two stainless steel electrodes, then electrochemical impedance spectroscopy (EIS) was conducted. The *L* and *S* of CPE-P(50) are 292 μm and 0.785 cm^2 (the diameter of the CPE-P(50) film is 1.0 cm), respectively. From the obtained Nyquist plot (as seen in Figure S3.), the bulk resistance *R* is 14.9 Ω at 58°C, so the (58°C) calculated by equation (1) is 2.5 mS cm^{-1}.

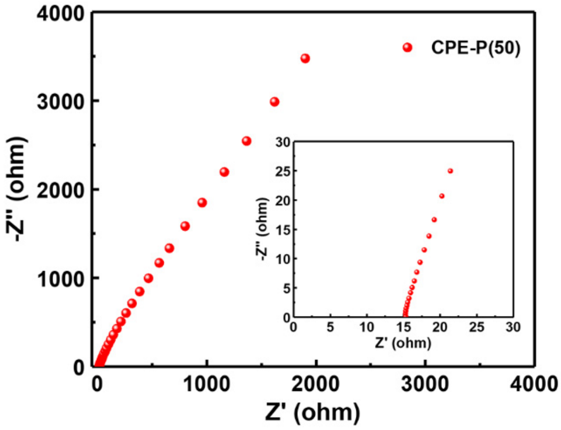

Figure S3. The Nyquist plot of SS |CPE-P(50)| SS cell at 58°C.

4. Lithium transference number

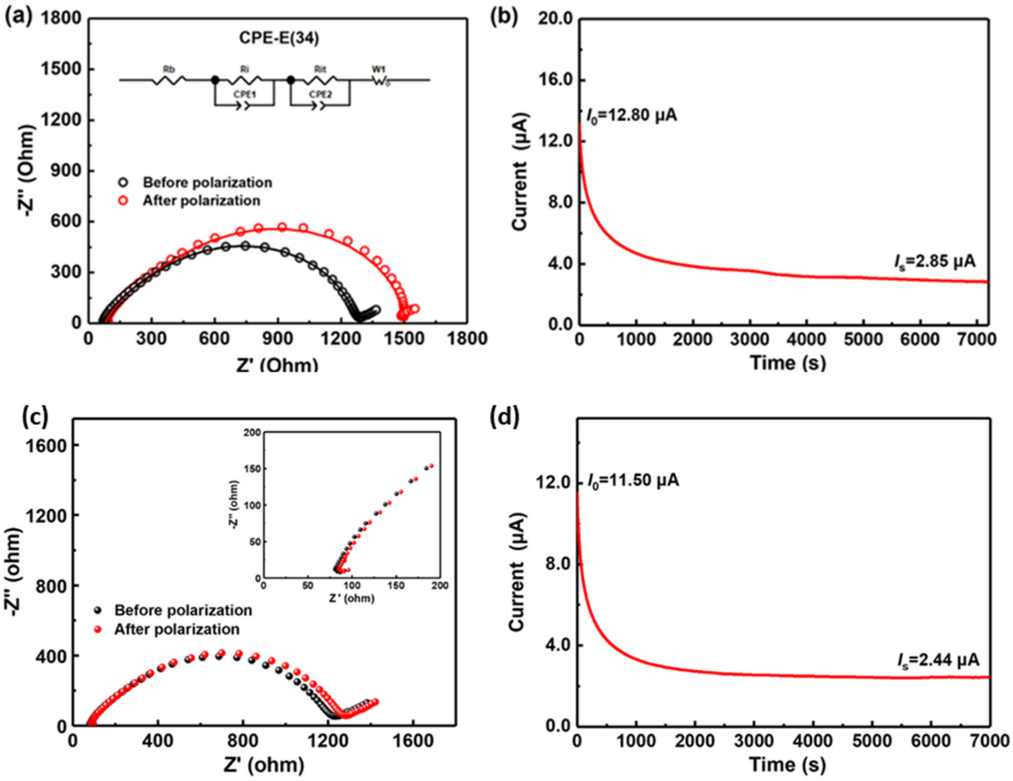

Figure S4. Nyquist plots before and after polarization and fitting curves (solid curves) (a) and the polarization curve (b) of the Li|CPE-E(34)|Li cell, and Nyquist plots before and after polarization (c) and the polarization curve (d) of the Li|CPE-P(42)|Li cell with a potential step of 20 mV at 20°C.

5. Polarization test of the lithium symmetric cells

The galvanostatic polarization test of Li|CPEs|Li cells were performed at room temperature under 0.03mA cm^{-2} to evaluate the ability to inhibit the growth of lithium dendrites, the obtained results are shown in Figure S5. The sudden decrease of the cell voltage to zero indicating the short circuiting of the cell. The short-circuit times of the two types of CPEs decrease gradually with increasing ionic liquid content. The short-circuit time of CPE-P(ω) is higher than that of CPE-E(ω).

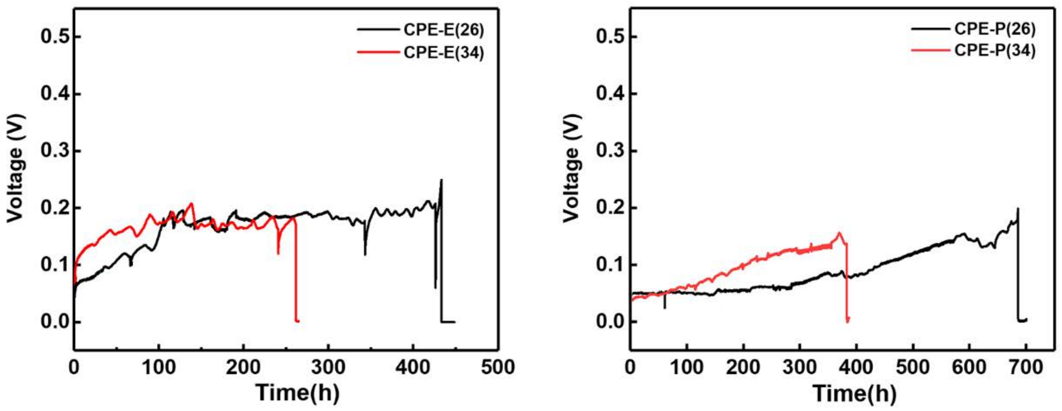

Figure S5. Galvanostatic polarization curves of different lithium symmetric cells under 0.03 mA cm^{-2} current density at 20 °C.
